# Enhanced measures to quantify gait and balance impairment in degenerative cervical myelopathy: A prospective cohort study

**DOI:** 10.1016/j.xnsj.2026.100904

**Published:** 2026-05-28

**Authors:** Khadija Soufi, Omar Ortuno, Tiffany Chu, Jose A. Castillo, Alan Harris, Muhammad Sulman, Kee D. Kim, Richard Price, Yashar Javidan, Rolando Roberto, Hai V. Le, Safdar Khan, Eric O. Klineberg, Sukhvinder Kalsi-Ryan, Michael G. Fehlings, Allan R. Martin

**Affiliations:** a Spinal Cord Injury, Function and Imaging Laboratory, Department of Neurosurgery, University of California, Davis, California, United States; b Department of Neurosurgery, University of California, Davis, California, United States; c Creighton University School of Medicine, Omaha, NE, United States; d Department of Orthopedic Surgery, University of California, Davis, CA, United States; e Department of Orthopedic Surgery, McGovern School of Medicine, Houston, TX, United States; f Spinal Cord Injury Center and Department of Neurology and Neurophysiology, Balgrist University Hospital, University of Zurich, Zurich, Switzerland; g KITE Research Institute, Toronto Rehabilitation Institute, University Health Network, Toronto, ON, Canada; h Department of Physical Therapy, UHN2, University of Toronto, Toronto, Ontario, Canada; i Department of Surgery, University of Toronto, Toronto, Ontario, Canada

**Keywords:** Degenerative, Cervical, Myelopathy, Gait, Balance, Neurological, Functional, Kinematics, Outcomes

## Abstract

**Background:**

Gait and balance impairment are primary symptoms of degenerative cervical myelopathy (DCM), but practical measures are lacking leading to delays in diagnosis and intervention. This study evaluated numerous gait and balance measures for their clinical and research utility.

**Methods:**

A prospective cohort study compared DCM and healthy subjects across several tasks: self-paced and fast-paced gait, tandem gait, tandem stance, single-leg stance, and Romberg with eyes open and closed. Tasks were scored manually and electronically using pressure mat data. Abbreviated Berg Balance (aBB) Scale, modified Japanese Orthopaedic Association (mJOA), and EuroQol 5-dimension were administered. Group comparisons used Mann–Whitney-U tests with Benjamini–Hochberg correction. Convergent validity was assessed with Spearman correlations to mJOA lower extremity (LE) scores. Subgroup analysis evaluated DCM patients with subjectively normal balance (DCM-SNB).

**Results:**

135 DCM patients and 110 age-matched healthy subjects were analyzed. DCM patients demonstrated impaired tandem gait, single-leg stance, aBB, velocity, gait stability ratio, and gait variability (p_adj_ < .001). Strongest correlations with mJOA LE were observed for self-paced walking velocity (r = 0.61) and aBB (r = 0.53). DCM-SNB patients demonstrated deficits in tandem gait and several fast-paced electronic gait metrics (p_adj_ < .05). The Modified 10-Step Tandem Gait Score showed impairment in DCM (p_adj_ < .001) and DCM-SNB (p_adj_ < .05), and moderate correlation with mJOA LE (r = 0.38).

**Conclusions:**

Gait and balance can be readily assessed in DCM patients using tandem gait, walking velocity, aBB, and single-leg stance. The modified 10-step Tandem Gait Score is a practical tool that demonstrates convergent validity and can detect subtle impairment prior to symptomatic recognition.

## Introduction

Degenerative cervical myelopathy (DCM) is a common disorder caused by spinal cord compression that leads to progressive motor, sensory, and autonomic impairments. This condition markedly reduces functional capacity and overall quality of life [1]. However, DCM is frequently misdiagnosed due to inadequate assessment tools and overlapping neurological and musculoskeletal conditions that delay appropriate intervention [2,3]. Prolonged compression can cause irreversible injury, leading to profound disability [4]. Cardinal neurological symptoms of DCM include upper extremity (UE) numbness and paresthesia, hand clumsiness, gait impairment, and urinary dysfunction (urgency or incontinence), while it is also associated with neck pain in a majority of patients [2,5]. Excluding pain symptoms, gait impairment is considered the highest priority for recovery by patients and if left untreated, can progress to dependence on assistive devices [3,6,7].

The various assessment tools for evaluation of gait and balance in DCM patients have several limitations and there is no consensus on optimal assessment. Common measures include the Nurick grade, Japanese Orthopaedic Association (JOA) score, the modified JOA (mJOA) score, Short Form 36 (SF-36) physical functioning score, and the Euroqol 5-dimension (EQ-5D) mobility score [[8], [9], [10], [11], [12], [13]]. However, these assessments are simple questionnaires without objectively defined levels and may not accurately characterize severity. A systematic review concluded that, although the mJOA is the most commonly used tool, there is no accepted ``gold standard'' for assessing severity of gait impairment or its progression [11]. Multiple studies recommend combining questionnaires like mJOA with objective, quantitative measures for a comprehensive evaluation [8,9,12,14]. Physical assessments such as the 30 m walk test [15], Berg Balance Scale (BB) [16], 10-step tandem gait test [13], and electronic gait analysis [17] have been investigated, but have not been thoroughly validated or gained clinical uptake.

In this study, we sought to address 3 objectives. First, we compared commonly used subjective clinical measures with standardized performance-based gait and balance assessments in patients with DCM. Second, we evaluated the performance and reliability of a pragmatic modified 10-step Tandem Gait Score derived from the previously described tandem gait test. Third, we investigated whether objective gait and balance metrics could detect subclinical functional impairment among DCM patients with subjectively normal balance (DCM-SNB) on mJOA score.

## Materials and methods

### Study design and subjects

This single-institution, prospective cohort study compared patients with DCM to healthy control subjects (HCS), and was approved by the University of California, Davis institutional review board (IRB #1723945-1). Patients referred to a neurosurgeon or orthopedic spine surgeon at UC Davis for DCM between August 2021 to June 2025 were evaluated and invited to participate if eligible, contingent on willingness and availability to complete an additional 1‑hour data collection session on the same day or during a return visit. Healthy subjects were recruited by convenience sampling, with efforts to balance age and sex to support multivariate analyses and serve as controls for DCM and other neurological conditions. Written informed consent was obtained from all participants, and data collection was conducted at the UC Davis Spinal Cord Injury, Function, and Imaging (SCIFI) Lab and/or the UC Davis Spine Center.

### Eligibility criteria

Inclusion criteria included English-speaking adults aged 18–90 years, while prisoners, pregnant women, and those unable to provide informed consent were excluded. Subjects were also excluded if they had nondegenerative causes of cervical myelopathy such as tumor, infection, cervical trauma, or rheumatoid arthritis. DCM subjects were required to have: (1) at least 1 symptom of upper extremity (UE) numbness/paresthesia, UE incoordination/clumsiness, subjective UE weakness, imbalance when walking, or autonomic dysfunction symptoms including urinary, sexual and bowel dysfunction; (2) at least 1 clinical sign including any hyperreflexia (UE or lower extremity [LE]), objective UE weakness, UE sensory loss, or gait ataxia; (3) imaging evidence of cervical cord compression, including circumferential compression, indentation, flattening, or torsion (from a lateral disc bulge) and (4) the absence of an alternative diagnosis that more likely explained symptoms. Findings were assessed qualitatively by experienced board certified spine surgeons. Imaging interpretation was performed in the context of a standard spine clinic evaluation and was not blinded to clinical presentation, reflecting real-world diagnostic practice. Subjects were examined for tremor, tongue fasciculations, pronator drift, rigidity, spasticity, rapid alternative movements, and finger-to-nose testing, specifically to identify other neurological conditions. Following data collection, all subjects were reviewed by 2 researchers (KS and ARM) to confirm inclusion, and subjects with an uncertain diagnosis were excluded from this analysis. However, we aimed to study a real-world cohort of DCM patients, which often have confounding comorbidities; thus, both DCM patients and healthy subjects were still considered eligible for inclusion if they had minor confounding conditions, (eg, joint replacement, carpal tunnel syndrome, lumbar radiculopathy), as long as these did not appear to be their primary cause of impairment. If a majority of gait and balance data were missing for a given patient then the patient was excluded from the study.

### Data collection

Extensive data collection included demographic data, medical history, and several questionnaires, such as the mJOA score, and EuroQol 5‑dimensions 5‑levels (EQ-5D-5L). All gait and balance assessments were performed using standardized scripted instructions delivered by trained study personnel. Tasks were performed with 1–2 research assistant next to the subject for safety, and tests were omitted if deemed unsafe and a score of 0 was assigned. No talking was allowed to avoid distraction throughout the testing procedures.

Balance testing included an abbreviated 9-item version of the Berg Balance (aBB) Scale (items 6–14), deemed most relevant for DCM (omitting simple tasks such as sit-stand). The following gait and balance tasks were evaluated using both manual scoring and electronic pressure mat analysis: self-paced and fast-paced walk (4 passes down a 5-meter walkway), tandem gait (1 pass, 5-meter walkway), tandem stance, Romberg test (with eyes open and closed), and single-leg stance (once on each foot). Participants performed these gait and balance maneuvers on a Protokinetics Zeno Walkway (Protokinetics LLC), an electronic pressure mat that provides automatic analysis of spatiotemporal gait parameters and calculation of center of pressure (COP) for tandem gait and each balance task. For self-paced walking, participants were instructed to walk at their normal comfortable pace for 4 lengths of the mat, starting and ending 1 meter beyond the end of the mat (to mitigate acceleration/deceleration). Fast-paced walking followed the same procedure but participants were instructed to walk at their fastest comfortable pace without running. In the tandem gait test, participants were asked to walk heel-to-toe along a taped midline, maintaining a pace of approximately 1 step per second.

### Manual scoring of gait and balance tasks

Each gait and balance test was manually scored by counting wobbles (substantial trunk or arm correction to maintain balance) and stumbles (an observable misstep or use of hands on a nearby wall or researcher) over a 10-second period for Romberg, tandem stance, and single-leg stance tests, for the first 10 steps for tandem gait, and over the duration of the walk for self-paced, and fast-paced walking. Single-leg stance scoring was averaged over left and right trials. We tested a variety of scoring schemes for each of the gait and balance tasks that used different weighting schemes of stumbles and wobbles. A 5-point ordinal scale was created for each task to simplify scoring. The scoring scheme of the previously reported 10-step tandem gait test was used as a model but was modified to add additional levels (Table 1) [13].

**Table 1:** Scoring rubric of the modified 10-step Tandem Gait Score.

JOA LE subscore	mJOA LE subscore	10-step Tandem Gait Score (Yoo et al. [13])	Modified 10-step Tandem Gait Score	Other gait/balance test scoring
4: Normal	7: Normal	4: Normal (takes 10 steps without sway)	5: Normal (takes 10 steps without wobbles/stumbles)	5: Normal (completes task without wobbles/stumbles)
3: Walks unaided, but slowly	6: Mild imbalance	3: Takes 10 steps, but unstable/swaying	4: Takes 10 steps, with wobbles but no stumbles	4: Completes task, with wobbles/sway
	5: Moderate imbalance		3: Takes 10 steps, 1-2 stumbles	3: Completes task, 1 stumble
2: Needs assistance on stairs	4: Must use handrail on stairs	2: Takes <10 steps before stumble	2: Takes 10 steps, 3 or more stumbles	2: Completes task, 2–3 stumbles
1: Needs walking aid	3: Needs walking aid	1: Takes ≤3 steps before stumble	1: Can walk with aid but unable to attempt safely	1: Completes task, >3 stumbles
0: Unable to walk	2: Some movement, unable to walk	0: Unable to walk	0: Cannot walk	0: Unable to complete
	1: No movement, some sensation			
	0: No movement or sensation			

JOA, Japanese Orthopaedic Association; mJOA, modified Japanese Orthopaedic Association; LE, lower extremity.

The table compares the scoring schemes of JOA LE subscore, mJOA LE subscore, 10-step Tandem Gait Score, our modified 10-step tandem gait score, and our scoring scheme for other gait/balance tests including self-paced walking, fast-paced walking, Romberg eyes-closed, Romberg eyes-open, tandem stance, and single-leg stance.

### Statistical analysis

Protokinetics Movement Analysis Software (PKMAS) was used to analyze gait and balance tasks. Self-paced and fast-paced walks were preprocessed by removing partial steps and identifying left and right footfalls. Software generated parameters included velocity, stride length, stride width, single support time (SST), double support time (DST), gait variability index (GVI), and gait stability ratio (GSR = SST/DST). For tandem gait and all balance tasks, COP was automatically calculated for all active pressure sensors on the electronic mat. Variability in COP was calculated as the standard deviation of the COP in the left-right (COP-LR) direction and the anterior-posterior (COP-AP) direction. Descriptive statistics were calculated for all parameters and are presented as mean ± standard deviation (SD). Spearman coefficients were calculated between all gait and balance parameters and several anchors (mJOA LE score, aBB), to assess for convergent validity and mJOA upper extremity (UE) score for divergent validity. A sub-analysis was also performed for the DCM-SNB subgroup of patients reporting normal balance (7/7) on mJOA LE. DCM, DCM-SNB, and DCM with subjective impaired balance (DCM-SIB) were each compared against healthy subjects using Mann–Whitney-U, and Kruskal-Wallis for DCM severity category comparison. All gait and balance tasks were video-recorded. Inter-observer reliability was assessed using intraclass correlation coefficients (ICC) calculated from independent scoring of recorded videos by 2 trained observers (KS and OO) who reviewed each task independently. ICC was calculated in a subset of patients with available video recordings, and selection was not influenced by disease severity. The Benjamini-Hochberg method was used to adjust p-values for multiple comparisons to keep the false discovery rate (FDR) less than 0.05. An “*” was used to denote a trend (unadjusted p < .05), “**” represents statistical significance (adjusted p < .05), and “***” indicates a highly significant result (adjusted p < .001). Adjustment was performed for testing of 107 hypotheses, but not for demographics and patient characteristics.

## Results

### Study population and baseline characteristics

About 135 DCM patients and 110 healthy subjects were included in the analysis. DCM patients (60.6 ± 14.2) were similar age to the HCS cohort (58.3 ± 17.0, p = .17) (Table 2). DCM patients trended towards elevated weight and BMI (both p = .07), while other characteristics were similar between groups. Based on mJOA score, 43% (n = 58) of DCM patients were categorized as mild, 40% (n = 54) as moderate, and 17% (n = 23) as severe. Comorbidities were broadly similar between DCM and HCS cohorts (all p ≥ .10). However, DCM patients had one or more relevant comorbidities more often than healthy controls (60.0% vs. 40.0%; p < .05).

**Table 2:** Participant demographics and baseline clinical characteristics.

	DCM (n = 135)	HCS (n = 110)	p-value
Age (y)	60.6 ± 14.2	58.3 ± 17.0	.17
Gender			
Male Female	44 (34%) 91 (66%)	33 (30%) 77 (70%)	.76
Height (inches)	65.6 ± 4.2	65.4 ± 4.0	.93
Weight (lbs)	178.7 ± 47.4	166.1 ± 42.4	.07
BMI (kg/m^2^)	29.2 ± 7.8	27.2 ± 6.6	.07
Race			
White Hispanic or Latino Asian Black or African Native American Other	109 (81%) 8 (6%) 6 (4%) 5 (4%) 2 (2%) 5 (4%)	83 (75%) 9 (8%) 10 (9%) 4 (4%) 1 (1%) 3 (3%)	.64
mJOA Score			
Normal (18) Mild ([15], [16], [17]) Moderate ([12], [13], [14]) Severe (<12)	0 (0%) 58 (43%) 54 (40%) 23 (17%)	70 (64%) 37 (34%) 3 (3%) 0 (0%)	<.001
Relevant comorbidities			
Neurological			
Lumbar stenosis/radiculopathy	14 (10%)	8 (7%)	.5
Peripheral neuropathy	7 (5%)	8 (7%)	.6
Carpal tunnel syndrome	16 (11%)	10 (9%)	.7
Chronic headaches	18 (13%)	10 (9%)	.42
Stroke	4 (3%)	3 (3%)	1
Other neurological disorders	8 (6%)	3 (3%)	.35
Musculoskeletal			
Hip/Knee arthroplasty	4 (3%)	2 (2%)	.69
Arthritis	25 (18%)	14 (13%)	.29
Other musculoskeletal disorders	12 (9%)	6 (5%)	.46
Endocrine			
Diabetes mellitus	17 (12%)	9 (8%)	.4
Patients with ≥1 comorbidities	84 (60%)	44 (40%)	<.05

BMI, body mass index; DCM, degenerative cervical myelopathy; HCS, healthy control subject; mJOA, Modified Japanese Orthopaedic Association Scoring System.

This table compares the demographics and characteristics between DCM and HCS participants. Data are presented as mean ± standard deviation and count (%). Displayed p-values are unadjusted.

### Comparison of subjective and performance-based gait and balance measures

• Manual Assessment of Gait and Balance

**Fig. 1:**
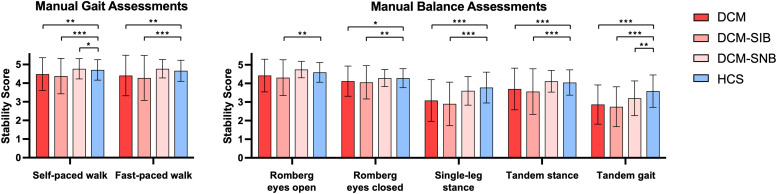
Manual gait and balance assessments comparing DCM patients, including subgroups DCM-SIB and DCM-SNB, with healthy control subjects (HCS). Tests performed include self-paced walk, fast-paced walk, Romberg eyes open/closed, single-leg stance, tandem stance, and tandem gait. Data are presented as mean ± SD. An “*” was used to denote a trend (unadjusted p < .05), “**” represents statistical significance (adjusted p < .05), and ‘***’ indicates a highly significant result (adjusted p < .001) when compared with the HCS cohort. DCM, degenerative cervical myelopathy; DCM-SIB, subjective impaired balance; DCM-SNB, subjective normal balance; HCS, healthy control subjects; SD, standard deviation.

**Fig. 2:**
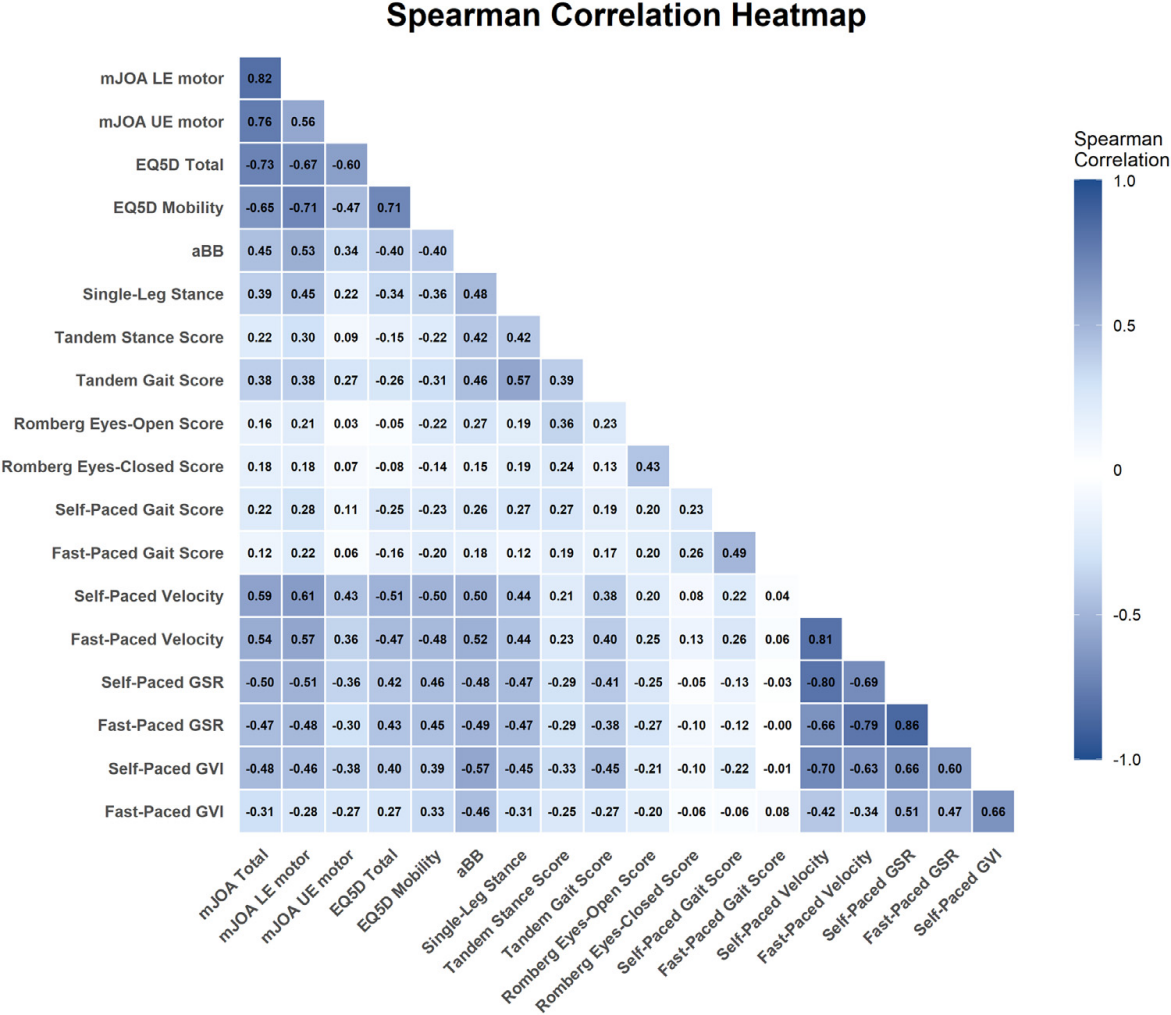
Heat map displaying correlations of electronic self-paced (left) and fast-paced (right) gait parameters versus clinical outcomes and cross-correlations. Spearman correlation coefficients between electronically measured self-paced gait metrics (velocity, stride length, stride width, single-support time, double-support time, gait stability ratio, gait variability index) and validated outcome measures (mJOA total, mJOA LE subscore, EQ-5D total, EQ-5D mobility, Berg Balance total). Darker shading represents stronger correlations (higher absolute r-value), lighter shading represents weaker correlations. Numeric r-values are shown in each cell. mJOA, Modified Japanese Orthopaedic Association; LE, lower extremity; EQ-5D, EuroQol 5-Dimension survey.

**Fig. 3:**
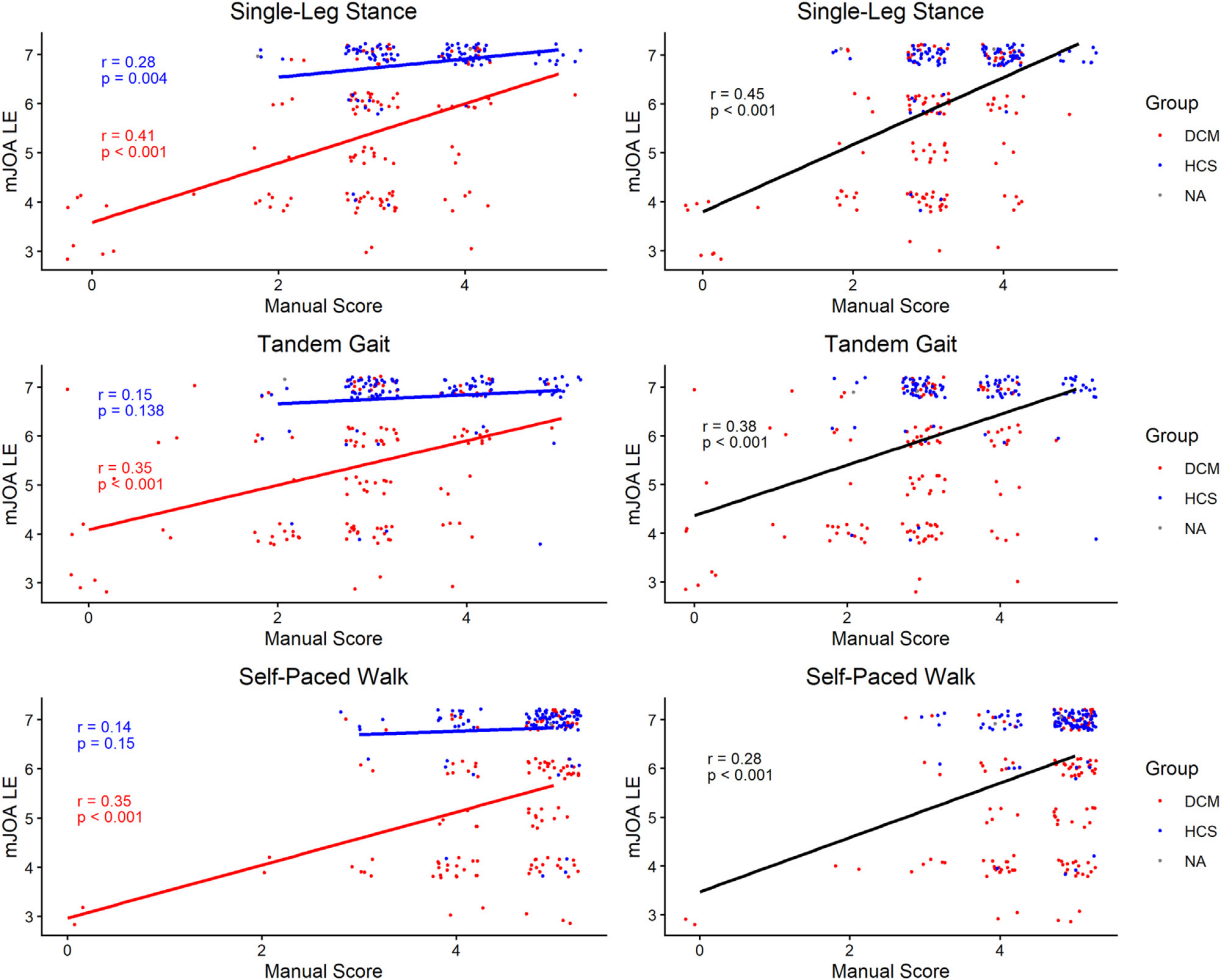
Correlations of manual gait tests with mJOA lower extremity subscore. Scatter plots display the relationship between mJOA LE and manual scores for (A) single-leg stance, (B) tandem gait, and (C) self-paced walk. Data points for DCM patients (red) and healthy control subjects (HCS, blue) are shown with separate linear regression lines and accompanying Spearman r and p-values for each group. mJOA LE, Modified Japanese Orthopaedic Association lower extremity subscore; DCM, degenerative cervical myelopathy; HCS, healthy control subjects. An “*” was used to denote a trend (unadjusted p < .05), “**” represents statistical significance (adjusted p < .05), and “***” indicates a highly significant result (adjusted p < .001) when compared with the HCS cohort.

Among various scoring schemes for manual gait and balance assessments (Supplementary Table S1), we selected 5-point scales (Table 1) that demonstrated good results and were utilized for the remainder of the analysis. DCM patients demonstrated impaired function on tandem gait, single-leg stance, and aBB (p_adj_ < .001), in addition to self-paced walking (p_adj_ < .05) (Table 3). Mild, moderate, and severe subgroups of DCM patients significantly differ in their performance on self-paced walking, fast-paced walking, tandem gait, and single-leg stance (Table 4). Tandem gait and single-leg stance showed stronger differences between DCM and healthy subjects than all individual components of aBB (Fig. 1, Supplementary Table S5). In terms of convergent validity, self-paced walking velocity had the strongest correlation with mJOA LE (r = 0.61), followed by fast-paced velocity (r = 0.57), aBB (r = 0.53), single-leg stance (r = 0.45), and tandem gait (r = 0.38, Table 5, Supplementary Tables S2 and S3). Representative scatterplots illustrating these correlations between mJOA LE and manual gait scores are shown in Fig. 3. Tandem gait, walking velocity, and single-leg stance all showed moderate convergent validity with aBB as an alternative anchor (r = 0.46, 0.50, and 0.48, respectively; Fig. 2, Supplementary Tables S2 and S3). Tandem gait, single-leg stance, and aBB also demonstrated divergent validity, showing weaker correlations with mJOA UE (r = 0.27, 0.22, 0.34, respectively; Fig. 2).

**Table 3:** Manual gait and balance assessments.

Assessment	DCM (N = 135)	DCM-SIB (N = 100)	DCM-SNB (N = 35)	HCS (N = 110)
Self-paced walking balance†	4.5 ± 0.9‡	4.4 ± 1.0§	4.8 ± 0.5*	4.7 ± 0.5
Fast-paced walking balance†	4.4 ± 1.1‡	4.3 ± 1.2§	4.8 ± 0.5	4.7 ± 0.6
Romberg eyes-open	4.4 ± 0.9	4.3 ± 1.0‡	4.7 ± 0.4	4.6 ± 0.5
Romberg eyes-closed	4.1 ± 0.8*	4.1 ± 0.9‡	4.3 ± 0.5	4.3 ± 0.5
Single-leg stance	3.0 ± 1.1§	2.8 ± 1.1§	3.5 ± 0.7	3.6 ± 0.8
Tandem stance	3.7 ± 1.1§	3.6 ± 1.2§	4.1 ± 0.6	4.0 ± 0.7
Modified 10-step Tandem Gait Score	2.9 ± 1.1§	2.7 ± 1.1§	3.2 ± 0.9‡	3.6 ± 0.9
Abbreviated Berg Balance (9-items)	32.2 ± 4.7§	31.2 ± 5.0§	35.1 ± 1.5	34.7 ± 2.6

DCM, degenerative cervical myelopathy; DCM-SIB, DCM with subjectively impaired balance (mJOA lower-extremity score <7); DCM-SNB, DCM with subjectively normal balance; HCS, healthy control subjects.

Per-test sample size varies due to occasional missing or unsafe trials.

Each test is measured by an observer on a 5-point scale, except for Abbreviated Berg Balance (scored out of 36). Data are mean ± standard deviation. Comparison of balance and gait assessments among patients with DCM, subdivided into DCM-SIB and DCM-SNB, and HCS.

⁎ Used to denote a trend (unadjusted p < .05).

† Self-paced and fast-paced walking are reported using the 5-point manual balance scale.

‡ Represents statistical significance (adjusted p < .05).

§ Indicates a highly significant result (adjusted p < .001).

**Table 4:** Manual gait and balance assessments.

Assessment	DCM-mild (N = 58)	DCM-moderate (N = 54)	DCM-severe (N = 23)	p-value
Self-paced walking balance†	4.7 ± 0.5	4.4 ± 0.7	4 ± 1.5	.03*
Fast-paced walking balance†	4.7 ± 0.5	4.3 ± 1	3.9 ± 1.9	.04*
Romberg eyes-open	4.6 ± 0.5	4.5 ± 0.6	3.9 ± 1.6	.16
Romberg eyes-closed	4.2 ± 0.5	4.2 ± 0.5	3.5 ± 1.5	.05
Single-leg stance	3.2 ± 0.9	3 ± 0.9	2.5 ± 1.3	.01*
Tandem stance	3.9 ± 0.8	3.7 ± 1	3.2 ± 1.7	.24
Modified 10-step Tandem Gait Score	3.2 ± 0.9	2.8 ± 1	2.3 ± 1.3	.01*

Comparison of balance and gait assessments among patients with DCM, subdivided into mild, moderate and severe based on mJOA severity scales.

⁎ Statistical significance (adjusted p < .05).

† Self-paced and fast-paced walking are reported using the 5-point Manual Balance Scale.

**Table 5:** Convergent and divergent validity.

Gait/Balance measure	Convergent (mJOA LE)	Divergent (mJOA UE)
	R	r
Single-leg stance	0.45†	0.22†
Modified 10-step Tandem Gait Score	0.38†	0.27†
Tandem stance	0.30†	0.09⁎⁎
Self-paced walk	0.28†	0.11⁎⁎
Fast-paced walk	0.22†	0.06⁎⁎
Romberg eyes open	0.21†	0.03⁎⁎
Romberg eyes closed	0.18*	0.07⁎⁎

Correlations between clinical gait/balance metrics and mJOA lower and upper extremity subscores among DCM and HCS cohorts (N = 245).

This table presents Spearman correlation coefficients (r) between quantitative gait and balance measures and the mJOA subscores for LE and UE function for DCM and HCS cohort.

⁎ Used to denote a trend (unadjusted p < .05).

⁎⁎ Represents statistical significance (adjusted p < .05).

† Indicates a highly significant result (adjusted p < .001).

### Inter-rater reliability of manual scoring

Inter-rater reliability of 2 assessors for each manual score was assessed in 88 DCM patients. The 5-point scoring system demonstrated excellent reliability for tandem gait (ICC = 0.98), single-leg stance (0.94), and fast-paced walk (0.93) (Table 6). All other gait/balance tasks demonstrated good reliability with ICC values of 0.75–0.88. ICC values were higher for counting stumbles than counting wobbles for all tasks except self-paced walk and Romberg eyes-open, which both had very low counts of stumbles.

**Table 6:** Inter-rater reliability of manual scoring of gait and balance in 88 patients with DCM.

Gait/Balance metric	ICC		
	Stumbles	Wobbles	Score
Self-paced walk	0.14	0.94	0.75
Fast-paced walk	0.97	0.88	0.93
Romberg eyes-open	0.36	0.83	0.85
Romberg eyes-closed	0.83	0.82	0.87
Tandem stance	0.95	0.80	0.88
Single-leg stance	0.82	0.54	0.94
Modified 10-step Tandem Gait Score	0.97	0.96	0.98

This table summarizes the ICC calculated from 2 raters 88 patients across 3 rating domains—stumbles, wobbles, and score on a 5-point scale—for multiple gait and balance assessments. ICC was calculated using a 2-way mixed-effects, single-measure, consistency model [ICC(3,1)].

• Electronic Gait Analysis and Balance Assessment

**Fig. 4:**
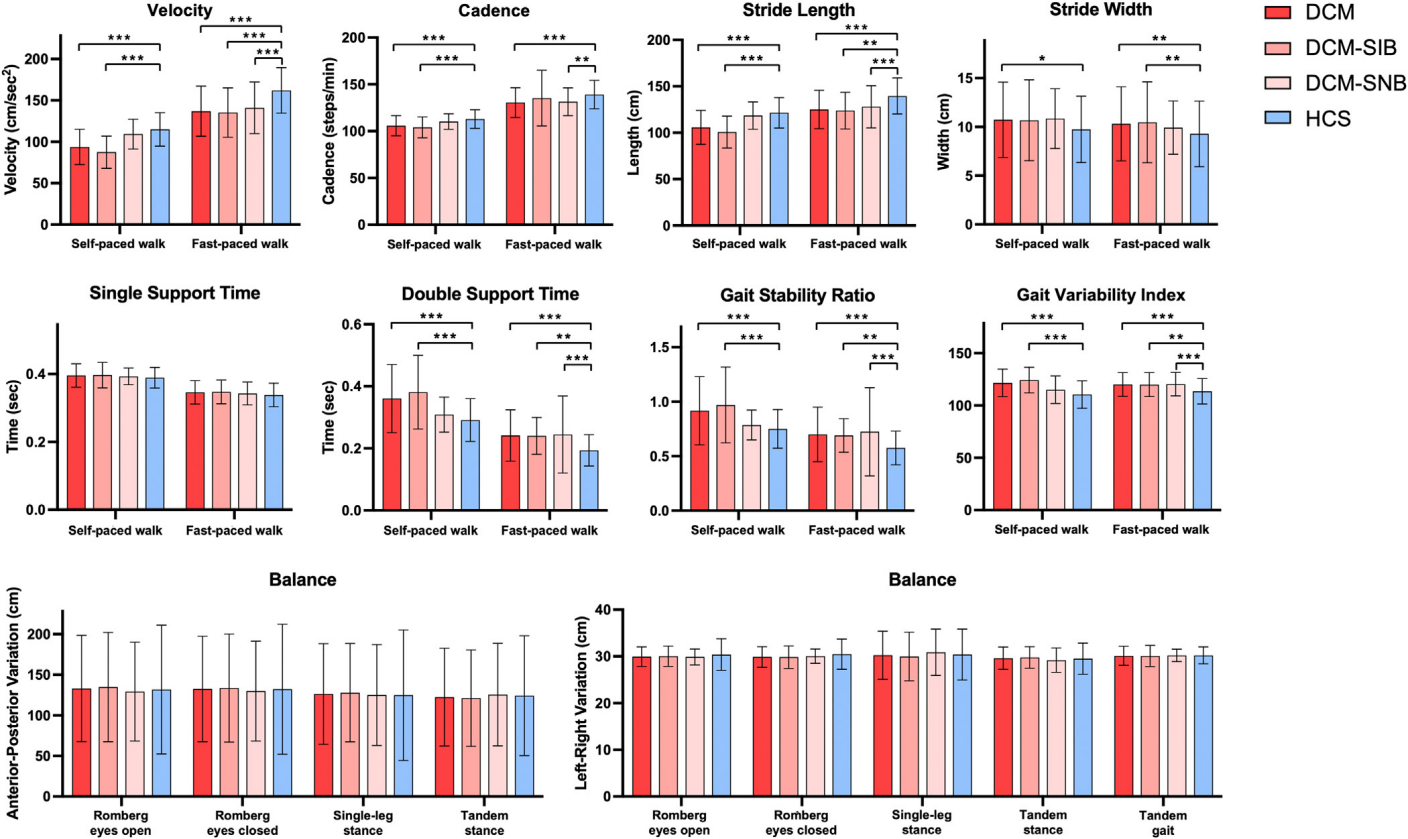
Electronic gait and balance assessments comparing DCM patients, including subgroups DCM-SIB and DCM-SNB, with healthy control subjects (HCS), measured using a pressure-sensing walkway. Parameters include velocity, cadence, stride length, stride width, single-support time, double-support time, gait stability ratio (GSR), gait variability index (GVI), and center-of-pressure variability in anterior–posterior (AP) and left–right (LR) directions. Data are presented as mean ± SD. Pairwise comparisons versus HCS: An “*” was used to denote a trend (unadjusted p < .05), “**” represents statistical significance (adjusted p < .05), and “***” indicates a highly significant result (adjusted p < .001) when compared with the HCS cohort. DCM, degenerative cervical myelopathy; DCM-SIB, subjective impaired balance; DCM-SNB, subjective normal balance; HCS, healthy control subjects; COP, center of pressure; AP, anterior–posterior; LR, left–right; SD, standard deviation.

Electronic gait analysis showed that DCM patients exhibited decreased velocity, cadence, stride length, and GSR, and increased double support time and GVI during both self-paced and fast-paced walking (all p_adj_ < .001; Fig. 4). DCM patients also showed trends toward greater stride width during both walking tests (p_raw_ < .05). Self-paced velocity and GSR showed the strongest convergent validity with mJOA LE (r = 0.61, −0.51, respectively; Fig. 2). When balance was assessed by COP variation on the electronic mat, DCM patients had greater variation in LR COP variability in single-leg stance (p_adj_ < .05), but no difference was noted in the AP direction. There were no differences between DCM and HCS patients in COP variability in AP or LR directions in other balance tests (Fig. 4).

### Performance of the modified 10-step Tandem Gait Score

The modified 10-step Tandem Gait Score demonstrated significant impairment in patients with DCM compared to HCS. Mean tandem gait scores were higher in healthy subjects than the entire DCM cohort (2.9 ± 1.1, p_adj_ < .001), DCM-SIB subgroup (2.7 ± 1.1, p_adj_ < .001) and DCM-SNB subgroup (3.2 ± 0.9, p_adj_ < .05) (Table 3). Tandem gait demonstrated moderate convergent and divergent validity, as reported above (Table 5), and excellent inter-rater reliability (Table 6).

### Subclinical impairment in DCM patients with subjectively normal balance (DCM-SNB)

Despite reporting normal balance on the mJOA questionnaire, the DCM-SNB subgroup demonstrated measurable deficits in balance measures. On manual assessments, tandem gait was significantly lower in DCM-SNB versus HCS (p_adj_ < .05). On electronic gait analysis, DCM-SNB patients demonstrated significant impairment during fast-paced walking across multiple spatiotemporal parameters compared with HCS, including reduced velocity (141.0 ± 31.2 vs. 162.1 ± 27.5 cm/s; p_adj_ < .001), shorter stride length (128.0 ± 22.8 vs. 139.6 ± 19.4 cm; p_adj_ < .05), lower cadence (131.4 ± 14.8 vs. 139.1 ± 15.2 steps/min; p_adj_ < .05), increased double support time (p_adj_ < .05), decreased gait stability ratio (p_adj_ < .05), and increased gait variability index (120.3 ± 11.3 vs. 113.5 ± 12.2; p_adj_ < .05). In contrast, self-paced walking parameters in DCM-SNB showed only a trend toward reduced GSR (p_raw_ < .05) without other significant differences, suggesting that fast-paced walking is more sensitive to subclinical gait impairment. No significant differences in COP variability were observed for any static balance task in the DCM-SNB subgroup (Supplementary Table S4).

## Discussion

This comprehensive prospective study is among the largest to evaluate gait and balance in DCM. We demonstrate that simple clinical measures, including tandem gait, single-leg stance, and timing of self-paced and fast-paced walking demonstrate statistically significant group-level differences between DCM and healthy subjects and show moderate correlation with established anchors, supporting their potential utility as adjunctive assessments. Electronic gait analysis of self-paced and fast-paced walking also revealed significant impairment in DCM across a wide range of parameters, including GVI and GSR, consistent with prior literature [10]. Notably, the subgroup of DCM patients reporting normal balance (DCM-SNB) demonstrated deficits in manually scored tandem gait and electronic assessment of fast-paced gait, suggesting that careful testing of gait and balance may identify neurological deficits in some patients before they perceive any impairment; this confirmed our *a priori* hypothesis and, importantly, indicates that subjective scales such as mJOA are insensitive to mild pathology. For pragmatic clinical use, we suggest clinicians may utilize the manually scored modified 10‑step Tandem Gait as a quick, accurate, reliable, and sensitive tool, while the addition of timed walking (eg, 30-meter walk test), single‑leg stance, and aBB provide a comprehensive assessment. Ultimately, our results have potential implications for achieving earlier diagnosis, more accurate characterization of severity, and improved surgical decision-making.

Our findings align with prior literature demonstrating decreased velocity, ataxic gait, and impaired balance in DCM [10,18]. Mandelli et al. reported slower gait speed, lower cadence, shorter stride length, greater step width, longer stride time, decreased single support phase (SMD, −0.68; 95%CI [−1.06; −0.29]; p = .011), and increased double support phase (SMD 0.84; 95%CI [0.35, 1.32]; p = .012), all consistent with our data [17]. Kalsi-Ryan et al. [10] found self-selected pace offers slightly superior results compared with fast-paced walking. In a small DCM cohort, Boerger et al. investigated a wide range of gait and balance measures and reported that DCM patients had reduced compensation during balance perturbation [18]. Lee et al. [19] investigated kinematics in 62 patients (some with and without subjective gait impairment) and demonstrated postoperative improvements in gait parameters, even in those without subjective complaints of gait dysfunction, albeit without comparison to healthy controls. Recent systematic reviews further emphasize that objective gait assessments complement subjective tools such as mJOA [14]. While responsiveness was not assessed in this study, prior work suggests gait velocity may be among the most sensitive markers of postoperative change.

Unfortunately, there is no true “ground truth” assessment to compare against when measuring gait and balance dysfunction in DCM. Historically, the Nurick Classification, a subjective and crude scale, emphasized gait alone for DCM severity asessment [20]. JOA and mJOA scores broaden the severity assessment by including upper and lower extremity motor, sensation, and bladder function [21,22]. More recently, mJOA has become widely accepted and was used to inform clinical practice guidelines (CPGs), with surgery recommended for moderate (mJOA 12–14) and severe (mJOA < 12) patients [23]. However, Nurick, mJOA, and JOA are highly subjective, without any physical confirmation of impairment; furthermore, mJOA and JOA have somewhat limited inter-observer reliability, with mJOA LE showing good but not excellent reliability (ICC = 0.83) in 1 study [24,25]. These scores depend on subjective reporting that may be biased by patient perception, and use features (eg, cane or handrail use) that are often confounded by age‑related comorbidities. These scales also use terms such as “mild” that are not clearly defined, and do not provide a clear level for intermittent symptoms. An alternative objective assessment for balance in DCM is the Berg Balance (BB) Scale, comprising 14 physical tests [8,9]. We opted to use an abbreviated 9-item version of the test, omitting 5 tasks that were not relevant to DCM (sitting to standing, standing to sitting, sitting unsupported, standing for 2 minutes, and transfers). The remaining 9-item tool performed well in our study but was more time-consuming (6–10 minutes) than individual tasks such as tandem gait or timed walking. A 30-meter walk test (30-MWT), measuring self-paced velocity, has also been reported as accurate and reliable [26]. However, a recent effort to develop a Core Measurement Set for DCM research found that no outcome measures, including JOA, mJOA, BB, and 30-MWT, achieved a COSMIN Category A recommendation, underscopring the need for improved assessment tools.

**Fig. 5:**
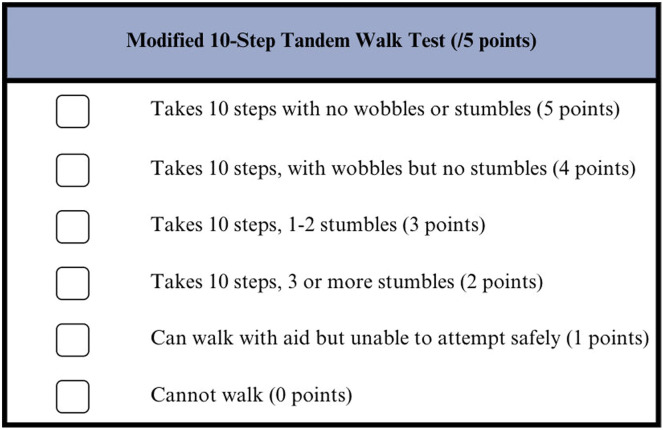
Modified 10-step Tandem Gait Test. This figure displays a modified scoring scheme, based upon the 10-step Tandem Walk Test proposed by Yoo et al. [13]. The scoring assesses stability during heel-to-toe (ie, tandem) walking based on the presence of wobbling/sway (any substantial trunk or arm correction made by the participant to maintain balance) or stumbles (an observable misstep or use of hands on a nearby wall or person to maintain balance). It includes 6 performance levels with corresponding checkboxes for clinicians or evaluators to record a participant’s performance out of 5 points, with lower scores represent increasing instability or inability to complete the task safely.

The 10-step tandem gait test was developed by Yoo et al. is a simple, objective adjunct to JOA, and its levels were created to reflect and validate JOA LE subscore (Table 1) [13]. We modified the scoring scheme to count stumbles during the 10 steps, rather than steps between each stumble, so that we could add additional levels to reflect a broader spectrum of severity in DCM. Yoo et al. described taking the better of 2 trials, but we designed our version to be performed only once in order to minimize time and learning effects [13]. We also considered a variety of alternative scoring schemes, including assigning scores for each stumble and wobble during tandem gait, but these had lower inter-observer reliability and were more cumbersome for the assessor, rendering them impractical. An important product of our study is the modified 10-step Tandem Gait Score, which is a straightforward tool to assess tandem gait that is sensitive to mild pathology, practical (less than 2 minutes), reliable, and shows convergent validity with mJOA LE and other anchors (Fig. 5).

However, it must be recognized that clinical score distributions often overlap between healthy and DCM populations, reinforcing the need for multiple assessments to achieve accuracy. The mean differences between DCM and HCS on our manual scoring of gait and balance were often less than 1 point (eg, tandem gait: 2.9 vs. 3.6), which is smaller than the smallest measurement increment. In fact, many healthy subjects exhibit wobbling or 1 stumble on tandem gait, limited the ability of this test to reliably classify individual patients. However, similar overlap was present with all of the measures assessed in this study, including mJOA and other questionnaires. The use of ordinal rating scales is also problematic, where the meaning of a 1-point change can vary substantially across the scale range. Importantly, tandem gait and other performance-based assessments are not intended to serve as standalone diagnostic instruments, but rather to complement subjective measures such as mJOA by providing objective, confirmatory data that can help inform clinical decision-making. The moderate correlations of tandem gait, single-leg stance, walking velocity, and aBB with established anchors support their use as part of a composite assessment strategy, consistent with prior recommendations to combine subjective and objective measures [9,10].

Electronic gait analysis yielded metrics that demonstrated larger effect sizes and may offer greater individual-level discrimination than ordinal manual scores. These include reduced velocity, shorter stride length, increased stride width, prolonged double support time, and increased variability, consistent with prior studies [10]. These patterns likely reflect disruption of corticospinal, spinocerebellar, and proprioceptive pathways. Increased stride width may represent a compensatory response to instability [19]. While electronic systems can detect subtle abnormalities, their cost, complexity, and time requirements limit routine clinical use [10,14]. In this study, electronic measures were primarily used as a reference, in the absence of a gold standard, to evaluate clinically feasible assessments.

To our knowledge, this is the first demonstration that DCM-SNB patients exhibit subclinical gait dysfunction. On manual testing, tandem gait was the only measure to reach significance in DCM-SNB, suggesting it is sensitive to detect dynamic balance deficits even in mild disease. Electronic gait analysis revealed a striking pattern: DCM-SNB patients appeared largely normal during self-paced walking but demonstrated significant impairment across multiple spatiotemporal parameters during fast-paced walking, including reduced velocity, shorter stride length, lower cadence, increased double support time, decreased gait stability ratio, and increased gait variability index (p_adj_ < .05). This suggests that the increased demands of fast-paced walking unmask subclinical corticospinal and proprioceptive deficits that are compensated for at comfortable walking speeds. Static balance measures did not differ between DCM-SNB and HCS, suggesting that dynamic rather than static assessments are more sensitive to early impairment. While these findings support the potential of objective testing to identify early dysfunction before patients perceive impairment, the clinical significance of group-level differences for individual patients is unclear, and external validation in independent cohorts is needed.

Limitations of this study include imperfect age-matching, potentially leading to bias in differences between groups, but this effect was likely minimal. The DCM cohort had a trend toward higher BMI, potentially indicating a lower level of general health and mobility, which could affect gait and balance measures negatively. Although we reported detailed subject characteristics (Table 2) to include potentially relevant comorbidities, differences in overall health status between DCM patients and healthy controls are difficult to fully account for and may contribute to observed functional performance differences. Our eligibility criteria allowed for patients with minor neurological and orthopedic conditions, in an effort to represent a real-world cohort of patients with DCM, but these conditions were not completely balanced between groups and could impact results. Imaging studies for DCM patients were reviewed by boad-certified spine surgeon to confirm diagnosis, but quantitative analysis of spinal cord compression or other imaging features was outside the scope of this study. The single-institution nature of the study could limit generalizability, and external validation is needed. Additionally, conversion of a continuous functional task into ordinal categories may introduce floor or ceiling effects that could limit sensitivity in detecting subtle performance differences. The full BB scale was not administered, limiting direct comparison with prior studies. The Modified 10-Step Tandem Gait Score represents a pragmatic adaptation of the previously described tandem gait test, but external validation is needed to ensure generalizability to routine clinical environments.

## Conclusion

This study demonstrates that patients with DCM exhibit significant impairments in gait and balance, even among those who report subjectively normal balance on standard clinical assessments such as the mJOA. The Modified 10-Step Tandem Gait Score is a simple, rapid, and pragmatic tool that can be performed in less than 1 minute without specialized equipment, offering objective data to supplement subjective scores such as mJOA and contribute to a more comprehensive assessment of disease severity. Berg Balance Scale, timed walking, and single-leg stance are also useful tests to provide a more comprehensive assessment. Quantitative analysis using an electronic pressure mat provides a highly detailed assessment of gait and balance, capturing subtle abnormalities across multiple spatiotemporal parameters and offering additional insight into the underlying pathophysiology of DCM. Our findings support the use of standardized manual assessments such as the Modified 10-Step Tandem Gait Score in routine clinical practice to facilitate earlier diagnosis, more accurate grading of severity, and better-informed management decisions for patients with DCM.

## Declaration of competing interests

The authors declare that they have no known competing financial interests or personal relationships that could have appeared to influence the work reported in this pape.

## Appendix Supplementary materials

Image, application 1
