# Supplementary material for: Enhanced measures to quantify gait and balance impairment in degenerative cervical myelopathy: A prospective cohort study
Source: N Am Spine Soc J. 2026 May 28;27:100904. doi: 10.1016/j.xnsj.2026.100904 (PMC13355517; doi:10.1016/j.xnsj.2026.100904)
Supplement: Supplementary file 1 [file mmc1.docx]

**Supplementary Materials**

**Table S1.** **Manual Measurements of Tandem Gait**. This table presents the mean tandem gait scores for patients with DCM and HCS across four different scoring schemes— Modified 10-Step Tandem Gait Test, 10-Step Tandem Gait Test, Weighted Stability Index – Version 1, and Weighted Stability Index – Version 2. Each scheme evaluates dynamic balance during tandem gait based on the frequency of stumbles and wobbles, with higher scores reflecting better gait stability. Correlation analyses were performed between each scoring scheme and standard clinical measures, including the total mJOA score, mJOA LE subscore, and Berg Balance Scale.

| **Scoring Scheme** | **DCM** | **HCS** | **p-value** | **Correlation to mJOA total** | **Correlation to mJOA LE** | **Correlation to Berg Balance** |
| --- | --- | --- | --- | --- | --- | --- |
| Modified 10-Step Tandem Gait Test (Soufi *et al.*)^12^ | 2.9 ± 1.1 | 3.6 ± 0.9 | < 0.001 | 0.38 | 0.38 | 0.46 |
| 10-Step Tandem Gait Test (Yoo *et al.*)^13^ | 2.8 ± 1.1 | 3.6 ± 1.1 | < 0.001 | 0.38 | 0.38 | 0.45 |
| Weighted Stability Index – Version 1 | 3.8 ± 1.6 | 4.6 ± 1.2 | < 0.001 | 0.36 | 0.20 | 0.60 |
| Weighted Stability Index – Version 2 | 3.8 ± 1.3 | 4.3 ± 1.1 | < 0.001 | 0.35 | 0.43 | 0.44 |

Modified 10-Step Tandem Gait Test (Soufi *et al.*)^12^ and 10-Step Tandem Gait Test (Yoo *et al.*)^13^ represent previously validated clinical scoring methods for tandem gait performance. Weighted Stability Index – Version 1 is a custom 0–10 weighted scoring scheme based on the frequency of stumbles and wobbles, where lower values indicate improved gait stability. Weighted Stability Index – Version 2 is a 0–30 weighted score calculated as $30-\frac{(5\times stumbles)+(1\times wobbles)}{3}$, emphasizing stumbles more heavily than wobbles. Higher scores correspond to better tandem gait performance and overall postural control.

DCM, degenerative cervical myelopathy; HCS, healthy control subject; LE, lower extremity; mJOA, Modified Japanese Orthopaedic Association Scoring System.

Data are presented as mean ± standard deviation.

**Table S2.** **Correlation between Manual Gait Assessments and Patient Outcome Measures.**

This table presents Spearman correlation coefficients (r) between quantitative gait and balance metrics and three clinical measures: the mJOA LE, the Berg Balance Scale, and the EQ-5D quality-of-life index.

| **Assessment** | **mJOA LE** | **Berg Balance** | **EQ5D** |
| --- | --- | --- | --- |
| Tandem gait | 0.38 | 0.46 | -0.26 |
| Tandem stance | 0.30 | 0.42 | -0.15 |
| Single-leg stance | 0.45 | 0.48 | -0.34 |
| Romberg eyes open | 0.21 | 0.27 | -0.05 |
| Romberg eyes closed | 0.18 | 0.15 | -0.08 |
| Self-paced walk | 0.28 | 0.26 | -0.25 |
| Fast-paced walk | 0.22 | 0.18 | -0.16 |

EQ-5D, EuroQol 5 Dimension survey; mJOA LE, Modified Japanese Orthopaedic Association Scoring System of the lower extremity.

**Table S3.** **Correlation between Electronic Self-Paced Gait Assessments and Patient Outcome Measures.** This table summarizes Spearman correlation coefficients (r) between quantitative gait parameters—velocity, GVI, and GSR—and three key clinical measures: the mJOA LE, Berg Balance Scale, and EQ-5D. These associations were analyzed separately for self-paced and fast-paced walking conditions to capture variations in performance across different gait speeds.

|  | **Assessment** | **mJOA LE** | **Berg Balance** | **EQ5D** |
| --- | --- | --- | --- | --- |
| Self-paced walk | Velocity | 0.61 | 0.50 | -0.51 |
|  | Gait Variability Index | -0.46 | -0.57 | 0.40 |
|  | Gait stability ratio | -0.51 | -0.48 | 0.42 |
| Fast-paced walk | Velocity | 0.57 | 0.52 | -0.47 |
|  | Gait Variability Index | -0.28 | -0.46 | 0.27 |
|  | Gait stability ratio | -0.48 | -0.49 | 0.43 |

EQ-5D, EuroQol 5 Dimension survey; mJOA LE, Modified Japanese Orthopaedic Association Scoring System of the lower extremity.

**Table S4.** **Electronic Gait and Balance Assessments.** This table summarizes mean (± SD) values for comprehensive gait and balance parameters across four groups: all patients with DCM, single-level involvement with DCM-SIB, DCM-SNB, and HCS.

| **Assessment** | **DCM** | **DCM-SIB** | **DCM-SNB** | **HCS** |
| --- | --- | --- | --- | --- |
| Velocity (cm/sec)  Self-paced  Fast-paced | 93.8 ± 21.14***  136.9 ± 30.2*** | 97.4 ± 19.5*** 135.3 ± 29.8*** | 109.3 ± 18.0  141.0 ± 31.2*** | 115.2 ± 20.0  162.1 ± 27.5 |
| Cadence (steps/min)  Self-paced  Fast-paced | 105.8 ± 10.6***  130.5 ± 15.9*** | 104.1 ± 11.2*** 135.3 ± 29.8 | 110.3 ± 8.3  131.4 ± 14.8** | 113.2 ± 10.0  139.1 ± 15.2 |
| Stride length (cm)  Self-paced  Fast-paced | 105.9 ± 18.1***  125.1 ± 20.6*** | 100.7 ± 17.2*** 123.9 ± 19.7*** | 118.5 ± 14.7  128.0 ± 22.8** | 121.5 ± 16.2  139.6 ± 19.4 |
| Stride width (cm)  Self-paced  Fast-paced | 10.7 ± 3.8  10.3 ± 3.8** | 10.7 ± 4.1 10.5 ± 4.2** | 10.9 ± 3.1  9.9 ± 2.7 | 9.7 ± 3.4  9.3 ± 3.4 |
| Stride time (sec)  Self-paced  Fast-paced | 1.2 ± 0.1***  0.9 ± 0.1*** | 1.2 ± 0.1***  0.9 ± 0.1*** | 1.1 ± 0.1  0.9 ± 0.1** | 1.1 ± 0.1 0.9 ± 0.1 |
| Single support time (sec)  Self-paced  Fast-paced | 0.4 ± 0.0  0.3 ± 0.0 | 0.4 ± 0.0  0.3 ± 0.0 | 0.4 ± 0.0  0.3 ± 0.0 | 0.4 ± 0.0  0.3 ± 0.0 |
| Double support time (sec)  Self-paced  Fast-paced | 0.4 ± 0.1***  0.2 ± 0.1*** | 0.4 ± 0.1*** 0.2 ± 0.1*** | 0.3 ± 0.1  0.2 ± 0.1** | 0.3 ± 0.1  0.2 ± 0.1 |
| Gait stability ratio  Self-paced  Fast-paced | 0.9 ± 0.3***  0.7 ± 0.3*** | 1.0 ± 0.3*** 0.7 ± 0.2*** | 0.8 ± 0.1*  0.7 ± 0.4** | 0.8 ± 0.2  0.6 ± 0.2 |
| Gait Variability Index  Self-paced  Fast-paced | 121.6 ± 13.2***  120.1 ± 11.3*** | 124.2 ± 12.3*** 120.0 ± 11.4*** | 115.0 ± 13.2 120.3 ± 11.3** | 110.4 ± 13.1  113.5 ± 12.2 |
| Romberg eyes open  AP COP variability  LR COP variability | 133.2 ± 65.3  30.0 ± 2.1 | 134.8 ± 67.3  30.0 ± 2.2 | 129.3 ± 60.9  30.0 ± 1.7 | 131.7 ± 79.3  30.4 ± 3.4 |
| Romberg eyes closed  AP COP variability  LR COP variability | 132.5 ± 65.0  29.9 ± 2.2 | 129.3 ± 60.9  29.8 ± 2.4 | 129.8 ± 61.6  30.0 ± 1.5 | 132.1± 80.1  30.5 ± 3.2 |
| Single-leg stance  AP COP variability  LR COP variability | 129.2 ± 64.3*  30.2 ± 5.1 | 127.7 ± 63.9**  30.0 ± 5.2 | 134.1 ± 66.6  30.9 ± 5.0 | 127.0 ± 81.2  30.4 ± 5.4 |
| Tandem stance  AP COP variability  LR COP variability | 122.4 ± 60.3  29.6 ± 2.4 | 129.3 ± 60.9  29.8 ± 2.3 | 125.6 ± 63.1  29.2 ± 2.6 | 124.3 ± 73.8  29.5 ± 3.3 |
| Tandem gait  LR COP variability | 30.1 ± 2.0 | 30.1 ± 2.2 | 30.2 ± 1.3 | 30.3 ± 1.8 |

AP, anterior-posterior; COP, center of pressure; DCM, degenerative cervical myelopathy; DCM-SIB, degenerative cervical myelopathy with subjective impaired balance (defined as a mJOA lower limb motor score < 7); DCM-SNB, degenerative cervical myelopathy with subjective normal balance (defined as a mJOA lower limb motor score of 7); HCS, healthy control subject; LR, left-right.

Data are presented as mean ± standard deviation.

* indicates statistical significance (p<0.05) when compared with the HCS cohort.

** indicates statistical significance (p<0.005) after correcting for multiple comparisons with Bonferroni adjustment when compared with the HCS cohort.

**Table S5**: Supplementary table with the individual components of the abbreviated berg balance score divided into total DCM group, DCM-SIB (mJOA lower extremity <7.0), DCM-SNB (mJOA lower extremity = 7) and HCS (healthy control). All p-values were compared to HCS

| **Assessment** | **DCM**  (N=135) | **DCM-SIB**  (N=100) | **DCM-SNB**  (N=35) | **HCS**  (N=110) |
| --- | --- | --- | --- | --- |
| Standing unsupported eyes closed | 3.8 ± 0.5** | 3.8 ± 0.6*** | 4.0 ± 0.2 | 4.0 ± 0.1 |
| Standing unsupported feet together | 3.8 ± 0.6** | 3.7 ± 0.7** | 3.9 ± 0.2 | 4.0 ± 0.2 |
| Reaching | 3.9 ± 0.5*** | 3.8 ± 0.5*** | 3.9 ± 0.2 | 3.9 ± 0.5 |
| Pick up object | 3.7 ± 0.8*** | 3.6 ± 0.9*** | 4.0 ± 0.0 | 3.9 ± 0.1 |
| Turning over shoulders | 3.4 ± 0.8*** | 3.3 ± 0.9*** | 3.9 ± 0.5 | 3.9 ± 0.4 |
| Turn 360 | 3.9 ± 0.5** | 3.9 ± 0.5** | 4.0 ± 0.0 | 4.0 ± 0.0 |
| Foot on step x 8 | 3.7 ± 0.7*** | 3.6 ± 0.7*** | 3.9 ± 0.4 | 4.0 ± 0.1 |
| Tandem stance | 3.1 ± 1.4 | 2.9 ± 1.5** | 3.9 ± 0.7 | 3.5 ± 1.3 |
| Standing 1 leg | 3.0 ± 1.2** | 2.8 ± 1.3*** | 3.6 ± 0.8 | 3.5 ± 1.1 |
| Total Berg Balance | 32.2 ± 4.7*** | 31.2 ± 5.0*** | 35.1 ± 1.5 | 34.7 ± 2.6 |
